# Comparison of condylar position after free fibular flap mandibular reconstruction using computer-assisted and traditional techniques

**DOI:** 10.1186/s12903-024-04203-y

**Published:** 2024-04-15

**Authors:** Yu Wang, Bowen Li, Juankun Liao, Yan Wang

**Affiliations:** 1 Department of Orthodontics, Stomatology Hospital of Guangzhou Medical University, 59th Huangsha Road, Guangzhou, Guangdong 510120 China; 2 Department of Oral and Maxillofacial Surgery, Sun Yat-sen Memorial Hospital, Sun Yat-sen University, 107th Yanjiang Xi Road, Guangzhou, Guangdong 510120 China; 3 Department of Stomatology, Sun Yat-sen Memorial Hospital, Sun Yat-sen University, 107th Yanjiang Xi Road, Guangzhou, Guangdong 510120 China

**Keywords:** Mandibular resection, Fibular flap reconstruction, Temporomandibular joint, Virtual surgical planning, Computer tomography

## Abstract

**Objectives:**

To compare the changes in condylar position after mandibular reconstruction with free fibular flap(FFF) and the differences between computer-assisted techniques and traditional methods on CT images.

**Methods:**

Thirty-four patients who underwent mandibular reconstruction with free fibular flap were selected according to the inclusion and exclusion criteria. In the 3D group, virtual surgical planning (VSP) with osteotomy cutting plate and placement guiding plate were used, while the traditional group underwent freehand reconstruction. The CT data of 68 temporomandibular joints (TMJs) were recorded before and immediately after surgery. The condylar position was evaluated by measuring the anterior space (AS), posterior space (PS) and superior space (SS), and the ln (PS/AS) was calculated according to the method proposed by Pullinger and Hollender.

**Results:**

In the patients included in the 3D group, the condyle on the ipsilateral side moved slightly backward; however, in the patients in the traditional group, the ipsilateral side moved considerably anteroinferior. No obvious changes on the contralateral side were noted. In the 3D group, 33% of ipsilateral condyles were in the posterior position postoperatively when compared with the preoperative position (13%). In the traditional group, the number of ipsilateral condyles in the anterior position increased from 4 to 10, accounting for 53% postoperatively. Contrary to the traditional group, the 3D group presented less condylar displacement on the ipsilateral side postoperatively.

**Conclusions:**

This study showed a decreased percentage of change in condylar position postoperatively when VSP was used. Virtual surgical planning improved the accuracy of FFF mandibular reconstruction and made the condylar position more stable.

## Introduction

Mandibular resection might be needed in various diseases, such as osteoradionecrosis, trauma, and cancer. Over the years, the free fibula flap (FFF) has become the preferred choice and gold standard for mandibular reconstruction [1, 2]. The primary goal of mandibular reconstruction is to achieve satisfactory morphology and function. Therefore, precise size and placement of the fibular segment, necessary osteotomies and native mandibular positioning are needed. The temporomandibular joint (TMJ) is a complex anatomical structure that plays an important role in mastication, speech and deglutition. Studies have shown that changes in condylar position after reconstruction can eventually lead to TMJ dysfunction, such as clicking, pain or condylar resorption [3]. Computer tomography (CT) is often used to evaluate condylar displacement after mandibular reconstruction because it can provide a clear image of the bones in the area of the TMJ. However, previous research on FFF mandibular reconstruction has mainly evaluated the operative effect, whereas changes in condylar position have rarely been studied.

Mandibular reconstruction with FFF remains complicated, and surgeons continue to try to simplify and improve the accuracy of the surgical procedure. Virtual surgical planning (VSP) is a computer-assisted tool that is used for preplanning osteotomies and positioning fibular segments, especially for patients who need multiple fibular segments. Furthermore, individualized reconstruction templates can be manufactured according to previous designs [4, 5]. Research has shown that compared to traditional techniques, VSP is advantageous in that it shortens the operative time and hospital stay, increases the precision of surgery and requires less reliance on surgeon experience [6, 7].

Hence, the aim of this study was to compare the changes in condylar position after mandibular reconstruction with free fibular flap by measuring the anterior space (AS), posterior space (PS), and superior space (SS) on CT images. Moreover, we compared the results of the computer-assisted technique and the traditional method. The authors hypothesized that condylar position would change after mandibular reconstruction with free fibular flap and that computer-assisted techniques would contribute to less displacement than traditional methods.

## Materials and methods

### Patients

In this retrospective study, we compared the clinical data of patients who underwent FFF mandibular reconstruction by computer-assisted 3D printing and conventional methods at the Department of Oral and Maxillofacial Surgery, Sun Yat-sen Memorial Hospital, Sun Yat-sen University from January 1, 2018, to February 28, 2023. All patients provided written informed consent, and the Ethics Committee of Sun Yat-sen Memorial Hospital, Sun Yat-sen University approved the study protocol (SYSKY-2023-394-01). Inclusion criteria: (1) patients who underwent FFF reconstruction of the unilateral mandible; (2) patients whose condyle was preserved; and (3) patients with preoperative and postoperative CT scans (postoperative CT was taken within 3 months after surgery). Patients with a pre- and postoperative unstable occlusion were excluded. Information on the patients’ sex, age, diagnosis, pathology, primary site, surgical technique and length of preserved condyle was documented.

### Surgical technique

In the 3D group, patients required VSP before surgery. The mandibular osteotomy site and fibula size were determined by VSP. Customized osteotomy cutting plate (for both mandible and fibular) and placement guiding plate were produced for each patient, as well as a pre-bent mandibular reconstruction titanium plate. In the traditional groups, mandibular osteotomy and FFF extraction were performed freehand according to the defect size. The titanium plate that was used for mandibular reconstruction was bent manually and fixed to the osteotomized fibula. The method of obtaining the FFF was the same in both groups. In both groups, mandibular reconstruction was completed by transferring the FFF to the bone defect and by performing microsurgical vascular anastomosis to connect the donor and recipient vessels.

### Data acquisition

Pre- and postoperative CT scanning were performed at the Institute of Radiology, Sun Yat-sen Memorial Hospital, Sun Yat-sen University. Imaging was performed with the patient’s head oriented in the midsagittal plane, perpendicular to the floor, and in the Frankfort plane, parallel to the floor. Patients bit in centric occlusion during exposure. We used a SIEMENS SOMATOM Sensation 64 multidetector row CT scanner (Siemens Medical Systems, Erlangen, Germany) to evaluate the change in condylar position. The device was set for 120 kVp, 200 mA, 0.5 mm slice thickness, and 0.5 s gantry rotation speed. In the 3D group, CT images were stored in DICOM format. Then, DICOM files were imported into Proplan CMF (Materialize, Leuven, Belgium) to reconstruct 3D virtual models of the maxillofacial skeleton and the bony fibula.

### Assessment of CT

**Fig. 1:**
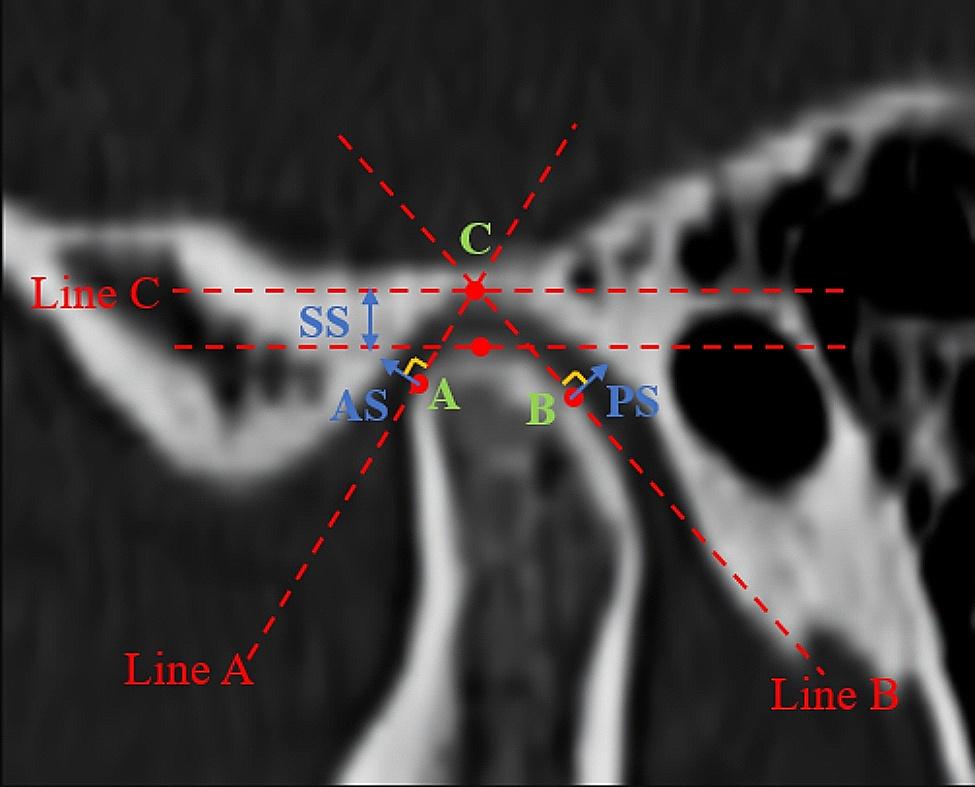
Landmarks and measurements of the condyle on the sagittal view. Line C is drawn parallel to the FH plane and intersects the glenoid fossa (point C). Point C is defined as the most superior aspect of the glenoid fossa. Lines A and B are tangent to point A and point B when drawn from point C. Points A and B are the most prominent anterior and posterior aspects of the condyle: AS (vertical distance from point A to the glenoid fossa), PS (vertical distance from point B to the glenoid fossa) and SS (vertical distance from point C to the condyle)

In this study, the reference plans, landmarks and measurements were defined as described in Table 1. We measured the space between the condyle and glenoid fossa on sagittal images, parallel to the midsagittal plane and passing through the center of the condyle. The specific methods were as follows. On the condylar midsagittal view, point C was defined as the most superior aspect of the glenoid fossa; line C was defined as parallel to the FH plane and intersecting the glenoid fossa; point A and point B were defined as the most prominent anterior and posterior aspect of the condyle; the lines tangent to point A and point B were drawn from point C, and they were defined as line A and line B. Subsequently, we measured the AS (vertical distance from point A to the glenoid fossa), PS (vertical distance from point B to the glenoid fossa) and SS (vertical distance from point C to the condyle) [8]. (Fig. 1) The spaces were calculated according to the method proposed by Pullinger and Hollender to assess the condylar position in the glenoid fossa [9]. The condylar positions were divided into three categories: (1) concentric if the ln(PS/AS) was at least − 0.25 but not greater than 0.25; (2) posterior if the ln(PS/AS) was less than − 0.25; and (3) anterior if the ln(PS/AS) was greater than 0.25.

**Table 1:** Reference landmarks, planes and measurements

Landmarks/planes/measurements	Definition on CBCT image
landmarks	
Porion	The superior surface of the external auditory meatus
Orbitals	The midpoint of the infra-orbital margin
Nasion	Nasofrontal suture at the midline
Basion	Middle point on the anterior margin of foramen magnum
A point	The most prominent anterior aspect of the condyle
B point	The most prominent posterior aspect of the condyle
C point	The most superior aspect of the glenoid fossa
Planes	
Frankfort horizontal (FH) plane	The plane that passes through the right porions and bilateral orbitales
Midsagittal plane	The plane that is perpendicular to the Frankfort plane and passes through the Na and Ba
Lines	
Line A	The line that is tangent to point A from point C
Line B	The line that is tangent to point B from point C
Line C	The line that is parallel to the FH plane and intersects the glenoid fossa
Measurements	
AS	The vertical distance from point A to the glenoid fossa
PS	The vertical distance from point B to the glenoid fossa
SS	The vertical distance from point C to the condyle

### Statistical analysis

Statistical analysis was performed using SPSS 26.0 (IBM, Armonk, NY, USA). Ten randomly selected samples were assessed for a second time at least two weeks after all the measurements were taken. The intraexaminer correlation coefficient (ICC) was used to evaluate the observer’s reliability. All ICC values were > 0.95, indicating good reliability. A paired T test was used to compare the change in condylar position after FFF mandibular reconstruction in each group. An independent T test was used to test the difference between different surgical techniques. A p value less than 0.05 was considered to indicate statistical significance.

## Results

### Demographics

This retrospective study included 34 patients. Patient data are shown in Table 2. The 3D group included 15 patients (8 males and 7 females; mean age, 30.47±15.18 years). The primary reason for mandible resection was ameloblastoma, which was present in 66.67% of the 3D group, and the pathology of 26.67% patients was squamous cell carcinoma. Only one patient had odontogenic keratocysts (6.66%). 8 on the left mandible and 7 on the right side. The average length of the remaining condyle was 19.22±2.93 mm. The traditional group included 19 patients (12 males and 7 females; mean age, 53.40±17.24 years). The pathology of 63.15% patients was squamous cell carcinoma, followed by ameloblastoma (26.32%) and osteoradionecrosis (10.53%). 7 on the left mandible and 12 on the right side. The average length of the remaining condyle was 19.80±5.26 mm.

**Table 2:** Characteristics and clinical data of the patients

Patients	Sex	VSP	Age(year)	Primary Site	Pathology	Condylar Preserved(mm)
1	Male	Yes	17	right	Ameloblastoma	21.1
2	Male	Yes	27	left	Ameloblastoma	17.2
3	Male	Yes	27	right	Ameloblastoma	18.8
4	Female	Yes	28	left	Ameloblastoma	25.2
5	Male	Yes	15	left	Ameloblastoma	21.5
6	Male	Yes	57	right	SCC	16.1
7	Male	Yes	18	right	Ameloblastoma	22.4
8	Female	Yes	19	right	Ameloblastoma	18.4
9	Male	Yes	29	right	OKC	17.6
10	Female	Yes	18	left	Ameloblastoma	14.9
11	Female	Yes	53	left	SCC	20.1
12	Female	Yes	51	left	SCC	17.2
13	Female	Yes	22	left	Ameloblastoma	19.6
14	Male	Yes	22	right	Ameloblastoma	22.6
15	Female	Yes	54	left	SCC	15.6
16	Male	No	56	right	SCC	16.0
17	Male	No	64	right	SCC	17.5
18	Male	No	73	right	osteoradionecrosis	11.8
19	Male	No	64	left	SCC	19.6
20	Female	No	66	right	SCC	22.6
21	Male	No	63	left	SCC	23.1
22	Female	No	25	left	Ameloblastoma	20.8
23	Male	No	61	right	SCC	20.7
24	Male	No	74	right	osteoradionecrosis	15.2
25	Male	No	27	left	Ameloblastoma	29.6
26	Female	No	20	left	Ameloblastoma	18.6
27	Female	No	55	right	SCC	15.2
28	Male	No	57	right	SCC	18.1
29	Male	No	67	right	SCC	20.7
30	Male	No	52	right	SCC	15.9
31	Male	No	27	left	Ameloblastoma	24.4
32	Female	No	68	right	SCC	33.6
33	Female	No	31	right	Ameloblastoma	17.0
34	Female	No	59	left	SCC	15.8

*Abbreviations* VSP, virtual surgical planning; OKC, odontogenic keratocyst; SCC, squamous cell carcinoma

### Changes in the temporomandibular joint space in the 3D group

**Fig. 2:**
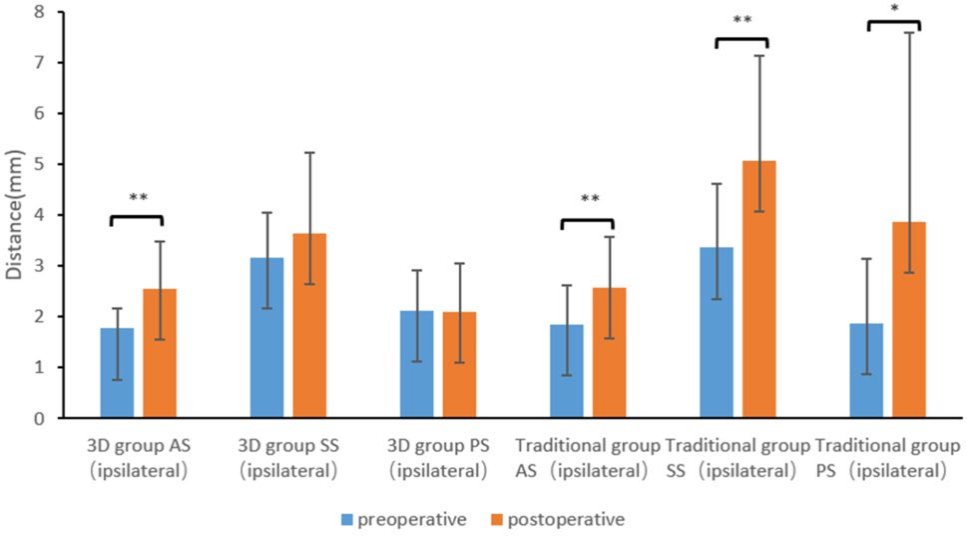
Pre- and post-operative distances of the AS, SS, and PS on the ipsilateral side. Significant differences were noted between the 3D and traditional groups (*p < 0.05, **p < 0.01)

Table 3 shows the results of the pre- and post-operative CT analysis of the temporomandibular joint space. A paired T test was used to assess the change in the condylar position. In the 3D group, only one indicator, AS on the ipsilateral side, was significantly different (*p* < 0.05). The other measurements showed no obvious change (*p* > 0.05). As presented in Fig. 2, the SS and PS on the ipsilateral side did not change significantly after surgery; however, the AS increased slightly, suggesting that the condyles moved slightly backward.

**Table 3:** Pre- and post-operative measurements of the temporomandibular joint space

Measurements	3D group			Traditional group					
	preoperative	postoperative	t value	p value	preoperative	postoperative		t value	p value
	(*n* = 15)	(*n* = 15)			(*n* = 19)	(*n* = 19)			
Anterior space (ipsilateral) (mm)	1.77±0.39	2.56±0.93	-4.077	0.001**	1.85±0.76	2.58±0.99	-3.700		0.002**
Superior space (ipsilateral) (mm)	3.16±0.88	3.65±1.58	-1.478	0.161	3.36±1.26	5.08±2.05	-4.201		0.001**
Posterior space (ipsilateral) (mm)	2.13±0.79	2.10±0.96	0.087	0.932	1.87±1.27	3.87±3.72	-2.412		0.027*
Anterior space (contralateral) (mm)	1.94±0.89	2.01±1.04	-0.455	0.656	1.79±0.69	1.99±0.67	-1.994		0.062
Superior space (contralateral) (mm)	3.10±0.78	3.02±0.88	0.375	0.712	3.11±1.13	3.16±1.21	-0.325		0.749
Posterior space (contralateral) (mm)	2.13±0.71	2.41±1.75	-0.665	0.517	1.82±1.13	1.90±1.55	-0.471		0.632

*The difference was significant (*p* < 0.05)

**The difference was significant (*p* < 0.01)

### Changes in the temporomandibular joint space in the traditional group

In the traditional group, all indicators of the temporomandibular joint space on the ipsilateral side showed obvious changes (*p* < 0.05), whereas that on the contralateral side did not change after surgery (*p* > 0.05). By comparing the pre- and postoperative measurements of the spaces, the SS and PS increased apparently on the ipsilateral side, which means that the condyles moved anteroinferiorly after surgery. (Fig. 2)

### Distribution of the pre- and post-operative condylar position

Table 4 shows the distribution of the condylar position in the two groups. In the 3D group, approximately 33% of ipsilateral condyles were in the posterior position postoperatively when compared with the preoperative position (13%). In the traditional group, the number of ipsilateral condyles in the anterior position increased from 4 to 10, accounting for 53% postoperatively. The above results were consistent with Fig. 2.

**Table 4:** Distribution of the pre- and post-operative condylar position

	3D group Condylar Position (ipsilateral)				Traditional group Condylar Position (ipsilateral)	
	preoperative	postoperative			preoperative	postoperative
	(*n* = 15)	(*n* = 15)			(*n* = 19)	(*n* = 19)
Anterior	5	2			4	10
Concentric	8	8			10	6
Posterior	2	5			5	3

### Comparison of condylar displacement between the 3D group and the traditional group

As presented in Table 5, the absolute difference in the pre- and post-operative SS and PS measurements on the ipsilateral side in the 3D group was smaller than that in the traditional group, and the difference was significant (*p* < 0.05). In contrast, on the contralateral side, the difference in all measurements was not statistically significant (*p* > 0.05). These results suggested that the displacement of the ipsilateral condyles in the 3D group was smaller than that in the traditional group.

**Table 5:** Measurements of condylar displacement between the 3D group and the traditional group

	3D group, *n* = 15	Traditional group, *n* = 19	t value	p value
Anterior space (ipsilateral) (mm)	0.79±0.75	0.84±0.76	-0.213	0.833
Superior space (ipsilateral) (mm)	0.86±1.04	1.91±1.59	-2.199	0.035*
Posterior space (ipsilateral) (mm)	0.88±0.76	2.58±3.18	-2.253	0.035*
Anterior space (contralateral) (mm)	0.44±0.35	0.34±0.34	0.876	0.388
Superior space (contralateral) (mm)	0.63±0.51	0.53±0.46	0.602	0.551
Posterior space (contralateral) (mm)	0.92±1.36	0.56±0.54	1.066	0.294

*The difference was significant (*p* < 0.05)

## Discussion

To date, it is common to use FFF to repair mandibular defects. Compared with traditional techniques, computer-assisted mandibular reconstruction with a FFF to repair mandibular defects has been increasingly used by surgeons in recent years [10]. To the authors’ knowledge, previous research on FFF mandibular reconstruction has mainly focused on the operative effect and accuracy of mandibular reconstruction. Few studies have evaluated the changes in condylar position. In this study, the authors compared the changes in condylar position on CT images after mandibular reconstruction with free fibular flap and the differences between computer-assisted method and traditional techniques. Displacement of the condyle appears three-dimensional, which means that it can be found in the midsagittal plane, horizontal plane and coronal plane; hence, it is complicated. Therefore, in this study, we analyzed the midsagittal plane specifically to achieve a clear and intuitive view of all measurements in this plane.

Many studies have shown that changes in condylar position after mandibular reconstruction with FFF might cause TMJ clicking, pain, disc displacement and perforation, limited mouth opening or condylar resorption [3, 5, 11]. Wei et al. analyzed the CT images of 16 patients’ TMJs pre- and postoperatively and found that the ipsilateral condyles moved anteroinferiorly immediately after surgery and then moved anterosuperiorly thereafter [12]. Saddam et al. examined the CBCT scans of 30 patients who underwent unilateral mandibular reconstruction to study their condylar positions and found that condylar position changed in a downward direction and became larger as time went on, whereas there were no significant differences in the anteroposterior direction [13]. In this study, the condylar position changed in the patients in both the 3D group and the traditional group after surgery. Especially in the traditional group, all the measurements (AS, SS, PS) on the ipsilateral side showed obvious changes. The mean SS increased from 3.36±1.26 mm to 5.08±2.05 mm, and the mean PS increased from 1.87±1.27 mm to 3.87±3.72 mm, indicating that the condyle moved anteroinferiorly. The causes of postoperative condylar displacement may be related to the following: first, the main reason of postoperative condylar displacement would be the inaccurate position and placement of bone segments during surgery, therefore, a 3D-printed replacing guide which designed during VSP would provide surgeons a more precise method when placing and fixing the bone segments; second, the balance among masticatory muscles has been broken due to surgical manipulation; third, if the length of the FFF is not sufficient, it is necessary to pull the condyle forward to compensate for the deficiency, therefore, design an appropriate length of fibula according to the mandibular defect during VSP is important ; fourth, intra-articular edema may occur when the proximal bone segment is manipulated; and fifth, the use of anesthetics and muscle relaxants during surgery results in condylar displacement [14].

Many studies have shown that condyles are commonly centered in the glenoid fossa, thus suggesting that change would be unlikely [15, 16]. On the other hand, one study found that the condylar position in asymptomatic volunteers was randomly distributed in the glenoid fossa [17]. This has always been a controversial belief. In this retrospective study, in both the 3D group and the traditional group, most ipsilateral condyles were in the concentric position preoperatively. We also found that in the 3D group, there were 3 less ipsilateral condyles in the anterior position after surgery, and the number of ipsilateral condyles in the concentric position did not change. In the traditional group, there were 6 more ipsilateral condyles in the anterior position, while the number of ipsilateral condyles in both the concentric and posterior positions decreased. The above data further confirmed that the condyle on the ipsilateral side in the patients in the 3D group moved slightly backward and largely anteroinferior in the patients in the traditional group.

VSP is a vital tool that is used for mandibular reconstruction. The 3D-printed cutting guide increases the precision of mandibular osteotomy. The advantages of VSP in mandibular reconstruction is as follows: improved bony apposition, decreased surgery time, fast fixation of bony segments, preservation of the TMJ position, improved functional results, decreased incidence of condylar displacement and improved surgeon comfort [18–20]. Bartier et al. compared the accuracy of FFF mandibular reconstruction between a 3D group and a traditional freehand group and found that VSP could help to improve surgical accuracy and mandibular symmetry [21]. Yu et al. investigated 29 patients with benign mandibular tumors who underwent unilateral mandibular reconstruction using FFF and found superior positioning in the computer-assisted group. They considered that the computer-aided design could guide condyle positioning and increase the accuracy of mandibular reconstruction [22]. However, some scholars have different beliefs. In the present study, the absolute difference in the pre- and post-operative SS and PS measurements on the ipsilateral side in the 3D group was smaller than that in the traditional group. These data showed a decreased percentage of change in condylar position postoperatively when VSP was used. This could be attributed to the use of customized osteotomy cutting plate and placement guiding plate for each patient. The above results further certified that VSP improved the accuracy of FFF mandibular reconstruction and made the condylar position more stable.

However, the present study had some limitations. First, this was a retrospective study, and the number of patients in the two groups was not equal after strict screening and exclusion. Second, in this study, the condylar position was only measured in the condylar midsagittal view, which did not allow consideration of displacement in the horizontal plane or coronal plane. Therefore, it is necessary to perform additional research in the future.

## Data Availability

The datasets used and analysed during the current study available from the corresponding author on reasonable request.
